# Design of a Bandgap Reference with a High PSRR and Strong Load-Driving Capability

**DOI:** 10.3390/mi17010050

**Published:** 2025-12-30

**Authors:** Meng Li, Lei Guo, Bin Liu, Lin Qi, Binghui He, Yu Cao, Jian Ren

**Affiliations:** School of information Science and Engineering, Shenyang University of Technology, Shenyang 110870, China; limeng_sut@163.com (M.L.); triplestone_g@163.com (L.G.); 13998284051@163.com (B.L.); qilin880821@126.com (L.Q.); aixleis@163.com (B.H.); cy18341622925@126.com (Y.C.)

**Keywords:** bandgap reference, high load-driving capability, high PSRR, low temperature coefficient

## Abstract

This paper introduces an enhanced bandgap reference (BGR) design, addressing the shortcomings of traditional circuits, such as significant temperature drift, limited power-supply rejection, and inadequate load-driving capacity. The proposed design incorporates a symmetric folded common-emitter–common-base BJT amplifier with MOS-assisted biasing, employed in the proposed BGR, enforcing branch voltage symmetry to effectively suppress intrinsic offset caused by structural mismatch. By reducing the amplifier input offset, the circuit achieves improved reference voltage stability, a lower temperature coefficient (TC), and an enhanced power-supply rejection ratio (PSRR). Additionally, a negative-feedback adaptive current-adjustment driver is implemented to dynamically adjust the output current in response to real-time load changes. This method bolsters the load-driving capability and maintains a stable reference output across varying load conditions. The circuit was simulated using a 0.18 μm BCD process, revealing that with a 3.3 V supply voltage, the BGR produces a stable output voltage of 2.5 V, with a TC of $2.372\times 10^{-6}$ °C^−1^. The simulated PSRR is −114.2 dB at DC and −62.07 dB at 1 kHz. Moreover, under a 3.3 V supply, sweeping the load capacitance from 0.1 μF to 100 μF demonstrates that the reference voltage remains consistently regulated at 2.5 V, confirming its excellent load tolerance and output stability.

## 1. Introduction

In recent years, as electronic products have continuously advanced in functionality and performance, the demands for precision, stability, and power efficiency in integrated circuit (IC) chips have become increasingly stringent. In particular, the rapid growth of ultra-low-power applications in energy-autonomous IoT sensor nodes, wearable devices, and medical implants has further heightened the requirements on voltage reference circuits [1,2]. As a fundamental building block in analog, digital, and mixed-signal systems, the voltage reference plays a critical role in digital-to-analog converters (DACs), analog-to-digital converters (ADCs) [3,4,5], DC–DC converters, and various sensor interfaces [6]. The accuracy and stability of the reference voltage directly affect overall system precision and long-term reliability. Therefore, designing a high-accuracy, high-stability, and power-efficient reference voltage source is of paramount importance.

In IC design, the BGR is commonly employed to provide a stable reference voltage, thanks to its excellent temperature stability and process portability. For optimal system performance, a BGR must display a high PSRR and a low TC [7,8,9]. However, in practical design and testing, discrepancies often arise between simulation and measurement results primarily due to resistor mismatches and material variations during fabrication. Typically, optimizing TC relies heavily on the precise matching of resistor ratios [10,11]. To address this, our work introduces a digitally controlled tunable resistor array, which calibrates resistor values across different process corners to effectively compensate for mismatches caused by process variations. This method corrects TC deviation and enhances the temperature stability of the reference voltage.

In a BGR circuit, the input offset voltage of the amplifier significantly impacts output accuracy [12]. This offset voltage directly affects the output by causing the amplifier’s input nodes to become unbalanced, which introduces reference voltage offset and increases temperature drift. As the temperature varies, the TC of the amplifier’s offset voltage exacerbates this error, undermining the BGR’s temperature stability and long-term precision [13,14]. To mitigate this issue, this paper introduces a low-offset amplifier architecture that integrates a folded common-emitter and common-base topology. This design achieves high gain and low input offset, thereby enhancing the output accuracy and PSRR of the BGR while preserving circuit stability.

The BGR voltage source developed in this work targets high-precision 4–20 mA transmitter systems [15,16]. Within these systems, the reference voltage is crucial for providing a stable supply to essential components like the 16-bit Σ–Δ ADC, bias current source, and oscillator, necessitating robust load-driving capability. To meet this need, we incorporated a negative-feedback adaptive current adjustment driver circuit into the BGR. This circuit automatically adjusts the output drive current in response to load variations, enhancing both load-driving capability and output stability. As a result, the output voltage remains stable and reliable under varying load conditions.

Implemented in a 180 nm BCD process, the proposed design offers a high-precision BGR with a low TC and strong load-driving capability. The circuit achieves excellent initial accuracy, an extremely low TC, and low static power consumption. It maintains stability across a wide load capacitance range from 0.1 μF to 100 μF, demonstrating performance comparable to an ultra-low TC, load-capacitance-free LDO regulator.

The paper is organized as follows. Section 2 compares the conventional BGR approaches with the proposed method. Section 3 details the operating principle of the proposed BGR circuit. Section 4 describes the operating principles and design methodologies of the amplifier, driver, and switchable resistor array. Section 5 presents simulation results for the circuit’s performance metrics. Finally, Section 6 summarizes the key findings.

## 2. Conventional BGR and Comparison with the Proposed Method

**Figure 1:**
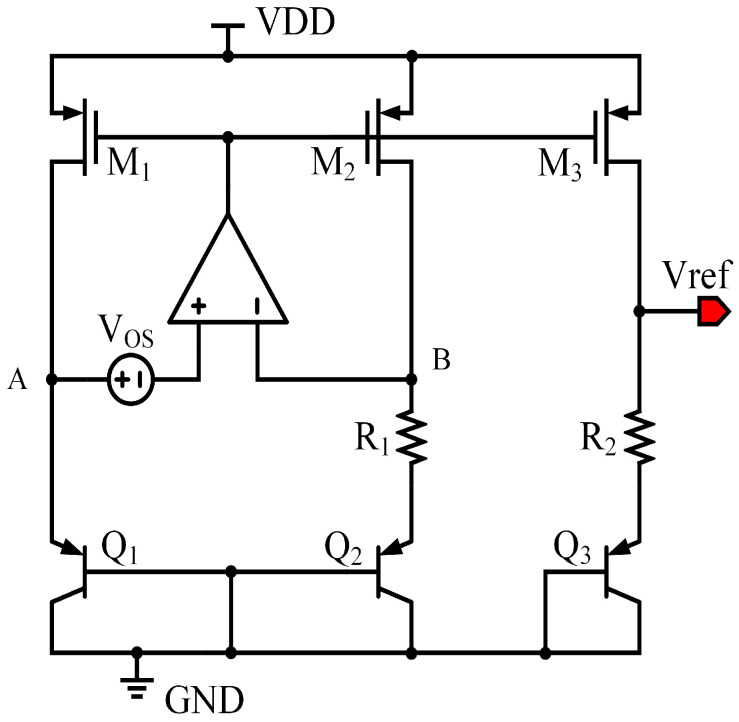
Conventional voltage mode BGR.

Figure 1 illustrates a conventional operational-amplifier-based Brokaw BGR. Under ideal conditions, the input offset voltage of the amplifier is neglected, and the amplifier forces the two input nodes (A and B) to the same potential, i.e., $V_{A}=V_{B}$. As a result, a well-defined base–emitter voltage difference $\Delta V_{BE}$ is generated between transistors $Q_{1}$ and $Q_{2}$, which produces a proportional-to-absolute-temperature (PTAT) current flowing through resistor $R_{1}$: $I_{PTAT}=\Delta V_{BE}/R_{1}$. The output bandgap voltage can then be expressed as $V_{BG}=V_{BE3}+I_{PTAT}R_{2}=V_{BE3}+\Delta V_{BE}R_{2}/R_{1}$, where $V_{BE3}$ is the base–emitter voltage of transistor $Q_{3}$.

When the input offset voltage of the amplifier is taken into account, the virtual short between nodes A and B is no longer ideal. Assuming an input offset voltage $V_{OS}$, the node voltages satisfy $V_{A}=V_{B}+V_{OS}$. This offset voltage directly perturbs the effective $\Delta V_{BE}$, leading to a modified PTAT current: $I_{PTAT}^{\prime }=\Delta V_{BE}+V_{OS}/R_{1}$. Accordingly, the bandgap output voltage becomes $V_{BG}^{\prime }=V_{BE3}+\Delta V_{BE}R_{2}/R_{1}+V_{OS}R_{2}/R_{1}$. From the above expression, the output voltage error caused by the amplifier input offset voltage can be written as $\Delta V_{BG}=V_{OS}R_{2}/R_{1}$. This result indicates that the amplifier offset voltage is directly amplified by the resistor ratio $R_{2}/R_{1}$ and appears at the bandgap output. As an illustrative example, if the resistor ratio $R_{2}/R_{1}$ is equal to 10, an amplifier input offset voltage of 1 mV will introduce an output voltage error of approximately 10 mV, which is non-negligible for high-precision BGR designs.

Compared with conventional operational-amplifier-based BGR architectures, the proposed design effectively addresses several inherent limitations, including sensitivity to amplifier input offset voltage, limited PSRR, and insufficient load-driving capability. Traditional BGRs rely heavily on the precision of the error amplifier, making their output voltage and temperature stability vulnerable to offset-induced errors and supply perturbations.

In this work, a digitally temperature-compensated BGR is adopted in conjunction with a low-offset operational amplifier, significantly suppressing the impact of offset voltage on the reference output and enabling a low TC. Furthermore, a symmetric folded common-emitter–common-base BJT amplifier with MOS-assisted biasing is employed in the proposed BGR, enforcing branch voltage symmetry to effectively suppress intrinsic offset caused by structural mismatch. By reducing the amplifier input offset, the circuit achieves improved reference voltage stability, lower TC, and enhanced PSRR. In addition, a negative-feedback adaptive current-adjustment driver is introduced to dynamically regulate the output current under varying load conditions, substantially improving the load-driving capability while maintaining a stable reference voltage. As a result, the proposed BGR achieves superior temperature stability, enhanced PSRR, and robust load regulation compared to conventional BGR designs.

## 3. BGR Circuit Structure

**Figure 2:**
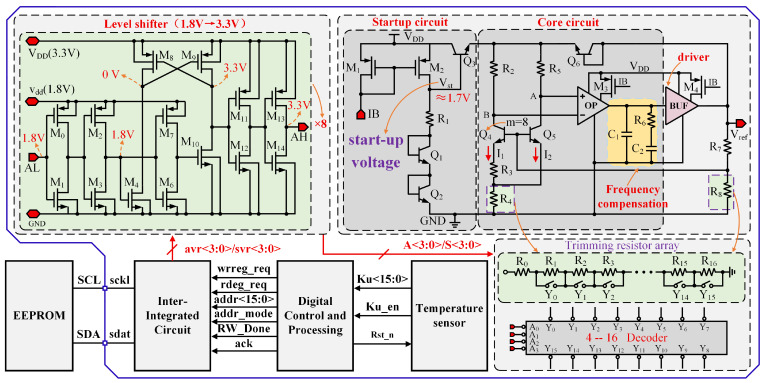
The proposed bandgap operation flowchart and the actual circuit.

Figure 2 illustrates the proposed BGR, which features a high PSRR, a low TC, and robust load-driving capability. The core bandgap circuit utilizes the Brokaw topology [17,18], and the complete design includes a startup circuit, a frequency compensation circuit, and a driver circuit.

In Figure 2, matched PMOS transistors $M_{1}$ and $M_{2}$ form a current mirror that reproduces the startup current and provides a stable bias reference for downstream circuitry. Resistor $R_{1}$ and transistors $Q_{1}$ and $Q_{2}$ constitute a bias network that stabilizes the circuit’s DC operating point. Transistor $Q_{3}$ serves as a startup trigger, with its base voltage $V_{st}$ acting as the pivotal node for the startup logic. When power is applied, a startup signal of approximately 1.7 V appears at $V_{st}$, switching on $Q_{3}$ and supplying initial current to $Q_{4}$ and $Q_{5}$. That initial current, amplified and driven by the amplifier and driver stages, drives the output voltage Vref upward. Vref is then fed back through resistors $R_{7}$ and $R_{8}$ to the bases of $Q_{4}$ and $Q_{5}$, thereby enabling the bandgap core. After startup completes, Vref settles at 2.5 V, which turns $Q_{3}$ off and effectively isolates the startup circuit, improving the BGR’s PSRR. To guarantee that $Q_{4}$ and $Q_{5}$ receive sufficient base voltage to conduct, Vref must be constrained by a minimum output-voltage limit.

The proposed BGR generates the PTAT current across resistor $R_{3}$, with the emitter area ratio of transistors $Q_{4}$ and $Q_{5}$ set to A_4_:A_5_ = 8:1. At steady state, the operational amplifier’s clamping action forces $V_{A}=V_{B}$. When $R_{2}=R_{5}$, currents $I_{2}$ and $I_{1}$ are equal. Under these conditions, the voltage across $R_{3}$ can be expressed as follows:

(1) $$\Delta V_{BE}=V_{BE5}-V_{BE4}=V_{T}\ln\left(\frac{I_{1}}{I_{2}}\;\cdot \;\frac{A_{4}}{A_{5}}\right)=V_{T}\ln8$$

The thermoelectric voltage $V_{T}$ is defined as the ratio of the thermal energy of an electron to its charge. The expression is given by $V_{T}=kT/q$, where *k* is the Boltzmann constant, $1.38\times 10^{-23}\;J/K$; *T* is the absolute temperature (K); and *q* is the electron charge, $1.602\times 10^{-19}\;C$. At room temperature (approximately 300 K), $V_{T}\approx 26\;\mathrm{mV}$.

Since the voltage difference $\Delta V_{BE}$ generates a current through resistor $R_{3}$, the PTAT current can be expressed as $I_{1}=V_{T}\ln8/R_{3}$. Since $I_{2}=I_{1}$, the voltage across $R_{4}$ is $2I_{1}R_{4}$. Therefore, the PTAT voltage across $R_{4}$ is

(2) $$V_{PART}=2I_{1}R_{4}=2\left(\frac{\Delta V_{BE}}{R_{3}}\right)R_{4}=\frac{2R_{4}}{R_{3}}\Delta V_{BE}=\frac{2R_{4}}{R_{3}}V_{T}\ln8$$

The base voltage of $Q_{5}$ in Figure 2 is

(3) $$V_{B5}=\frac{2R_{4}}{R_{3}}V_{T}\ln8+V_{BE5}$$

Vref is derived from $V_{B5}$ through a voltage divider formed by resistors $R_{7}$ and $R_{8}$.

(4) $$Vref=\left(1+\frac{R_{7}}{R_{8}}\right)\left(\frac{2R_{4}}{R_{3}}V_{T}\ln8+V_{BE5}\right)$$

As shown in Equation (4), the BGR output voltage Vref depends on the ratios $R_{4}/R_{3}$ and $R_{7}/R_{8}$. For a high-precision reference, both ratios must be adjustable [19,20]. With laser trimming, $R_{3}$ is the preferred target because adjusting $R_{3}$ also calibrates the current $I_{1}$ indirectly. When trimming is implemented, MOS transistors can be used as switches to control resistance; however, $R_{3}$ cannot be used. The MOS devices alter the TC of $R_{3}$, which makes the ratio $R_{4}/R_{3}$ temperature-dependent. Consequently, the $R_{4}/R_{3}$ ratio must be adjusted by trimming $R_{4}$. Because $R_{7}$ and $R_{8}$ have negligible influence on temperature behavior, either may be trimmed; in this design, $R_{8}$ is chosen.

## 4. Circuit Architecture and Implementation

### 4.1. Amplifier Circuit Design

**Figure 3:**
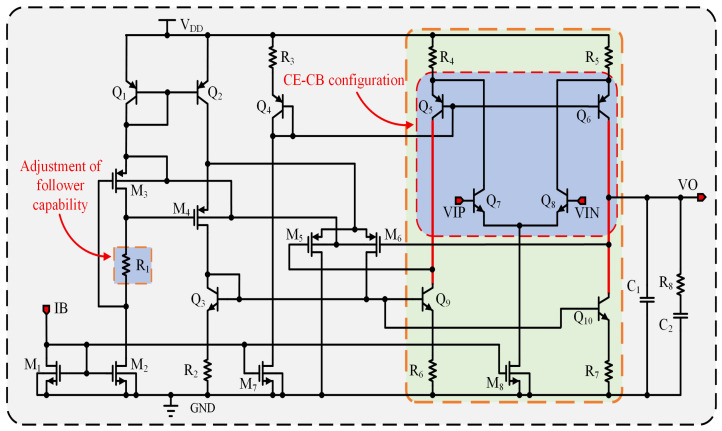
Amplifier circuitry.

The offset voltage of the amplifier is a crucial indicator for the accuracy of the bandgap reference [21,22,23]. Although previous analyses assumed perfect equality between VIP and VIN of the amplifier in a stable state [24], the reality is that due to the finite input impedance of the amplifier, there exists an offset voltage $V_{OS}$ resulting from the difference between the two inputs. This offset voltage $V_{OS}$ is temperature-dependent, thereby influencing the TC of the bandgap output [25]. Consequently, it is imperative for the amplifier in a bandgap circuit to exhibit low offset characteristics. Moreover, the BGR circuit in this design necessitates a substantial output current capacity and must remain stable within a load capacitance range of 0.1 μF to 100 μF, imposing additional demands on the frequency characteristics of the amplifier. Figure 3 depicts the comprehensive circuit schematic of the amplifier.

$Q_{5}$, $Q_{6}$, $Q_{7}$, and $Q_{8}$ form a folded common-emitter common-base amplifier. Bipolar transistors, compared to MOS transistors, offer superior matching characteristics, resulting in a smaller offset voltage [26]. $Q_{9}$ and $Q_{10}$, along with the differential pair $M_{5}$ and $M_{6}$, serve as the load. The gate voltage of $M_{5}$ is determined by the equation $V_{G5}=VO-|V_{GS6}|-|V_{GS5}|$. Since $M_{6}$ is identical to $M_{5}$, this means that $\left|V_{GS6}|=|V_{GS5}\right|$, and the gate voltage of $M_{5}$ changes with the output voltage VO.

When the supply voltage $V_{DD}$ falls, the emitter potential $V_{EB1}$ of $Q_{1}$ decreases, which lowers the source voltage of $M_{3}$, $V_{S3}=V_{DD}-V_{EB1}$. Therefore, the gate voltage of $M_{3}$ can be expressed as $V_{G3}=V_{S3}-\left|V_{GS3}\right|$. Including the voltage drop across resistor $R_{1}$, the drain voltage of $M_{3}$ becomes $V_{D3}=V_{G3}+V_{R1}$. Accorfingly, the source voltage of $M_{4}$ is given by $V_{S4}=V_{D3}-\left|V_{GS4}\right|$. By the same mechanism, the source voltages of $M_{5}$ and $M_{6}$ fall sequentially, and the chain finally determines the collector voltage of $Q_{9}$.

The gate voltage of $M_{5}$ does not completely track changes in the output voltage VO because, as VO increases, the source voltage of $M_{6}$ adjusts within its threshold voltage range: $VO-\left(V_{D3}-\left|V_{GS4}\right|\right)<\left|V_{th6}\right|$. Under these conditions, $M_{6}$ operates in the cutoff region, which weakens its load effect. Resistor $R_{1}$ plays a crucial role in regulating the collector voltage of $Q_{9}$. As $R_{1}$ increases, the source voltages of $M_{4}$, $M_{5}$, and $M_{6}$ also rise, which elevates the upper limit of the output voltage and prevents $M_{6}$ from prematurely entering cutoff. As a result, the gate voltage of $M_{5}$ increases in tandem with the output voltage.

**Figure 4:**
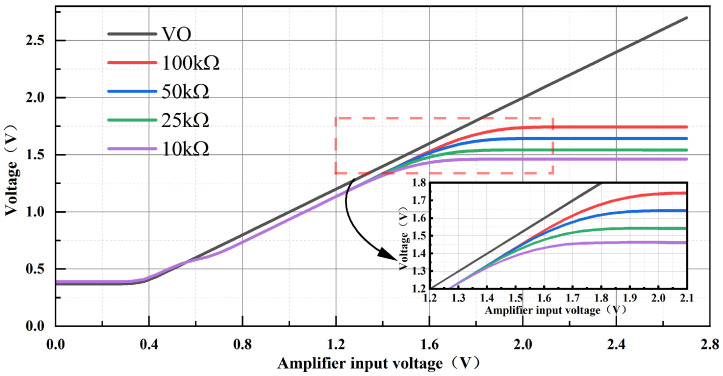
$Q_{9}$ Collector Voltage Variation with Output VO for Different $R_{1}$ Values.

This design offers an effective means to reduce offset voltage. In a folded common-emitter, common-base amplifier [27,28], the dominant offset arises from asymmetry between the upper and lower branches. To assess the design, we performed simulation analyses. As shown in Figure 4, when the input voltage is swept from 0.2 V to 0.7 V in DC analysis, the collector voltage of $M_{6}$ closely tracks VO, and this tracking improves as $R_{1}$ increases. Because the driver stage boosts the amplifier output by about 1.2 V, the amplifier output remains within the linear range when the driver output reaches approximately 2.5 V. Simulations also show that the amplifier can start up with an output voltage near 0.4 V, and the driver-stage boost ensures reliable startup of the BGR.

**Figure 5:**
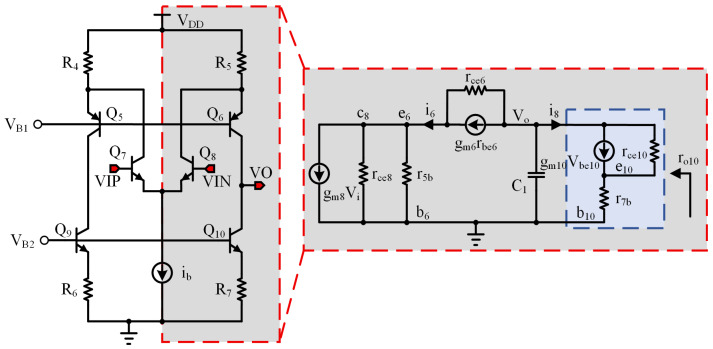
Core Section of the Amplifier and Half-Circuit Small-Signal Model.

To enhance readers’ understanding of each component’s function in the amplifier and the principles guiding their parameter design, a small-signal analysis of the circuit is essential. Figure 5 illustrates the core section of the amplifier, which is characterized by a symmetrical structure. Specifically, $Q_{5}$, $Q_{6}$, $Q_{7}$, and $Q_{8}$ constitute a folded cascode amplifier. A key feature of this amplifier is the inclusion of $M_{3}$, $M_{4}$, $M_{5}$, and $M_{6}$. Their primary role is to maintain electrical symmetry between the two branches of the folded cascode amplifier. This ensures that the collector voltage of $Q_{9}$ aligns with the output voltage VO, thereby enhancing both the gain stability and the common-mode rejection capability of the amplifier.

The amplifier’s core is symmetric, so a small-signal analysis may be performed on its half-circuit. From Figure 5, the half-circuit small-signal equivalent beginning at the input of transistor $Q_{8}$ is constructed. Here, $r_{5b}$ denotes the parallel combination of resistor $R_{5}$ and the base–emitter resistance of transistor $Q_{6}$ ($r_{be6}$), and $r_{7b}$ denotes the parallel combination of resistor $R_{7}$ and the base–emitter resistance of transistor $Q_{10}$ ($r_{be10}$). Because these two equivalent resistances are small relative to the other circuit impedances, only the high-impedance output node VO and its associated capacitance $C_{1}$ are retained in Figure 5. From this half-circuit small-signal model, the expression for the current $i_{8}$ is derived as follows:

(5) $$i_{8}=g_{m10}v_{be10}+\left(v_{0}-v_{e10}\right)g_{ce10}$$

Here, $g_{ce10}=1/r_{ce10}$. Since node $b_{10}$ is a small-signal ground, $v_{be10}=-v_{e10}$. Substituting this into Equation (5) yields $i_{8}=v_{o}g_{10}-\left(g_{m10}+g_{ce10}\right)v_{e10}$. Moreover, as can be seen from Figure 5, $v_{e10}=i_{8}r_{7b}$. It can be derived that

(6) $$r_{o10}=\frac{v_{o}}{i_{8}}=\frac{1+g_{m10}g_{ce10}r_{7b}}{g_{ce10}}=r_{ce10}+g_{m10}r_{7b}$$

Next, it is necessary to derive the relationship between $v_{e6}$ and $v_{o}$. As shown in Figure 5, $i_{8}=v_{o}g_{o10}$, where $g_{o10}=1/r_{o10}$. Additionally, $i_{8}=-i_{6}=-v_{o}g_{o10}$. Referring to Figure 5, the expression for the current $i_{6}$ can be derived as follows:

(7) $$i_{6}=g_{m6}v_{be6}+\left(-v_{o}v_{e6}\right)g_{ce6}$$

Since node $b_{6}$ is grounded, $v_{be6}=-v_{e6}$. Rearranging Equation (7) gives

(8) $$v_{e6}=\frac{g_{o10}+g_{ce6}}{g_{m6}+g_{ce6}}v_{o}$$

Next, the low-frequency gain is derived using Equation (8). Given that $i_{6}-g_{m8}v_{i}=v_{e6}g_{rb}$, where $g_{rb}$ represents the equivalent conductance of the parallel combination of $r_{ce8}$ and $r_{5b}$, we proceed by substituting $i_{8}=-i_{6}=v_{o}g_{o10}$ and Equation (8) into $i_{6}-g_{m8}v_{i}=v_{e6}g_{rb}$. This substitution yields

(9) $$-g_{m8}v_{i}=v_{o}g_{o10}+\frac{g_{o10}+g_{ce6}}{g_{m6}+g_{ce6}}v_{o}g_{rb}$$

Therefore, the expression for the amplifier gain can be obtained as

(10) $$A_{v}=\frac{v_{o}}{v_{i}}=\frac{-g_{m8}g_{m6}}{g_{o10}g_{m6}+g_{o10}g_{ce6}+g_{rb}g_{o10}+g_{rb}g_{ve6}}$$

For Equation (10), only the first term in the denominator is retained. Thus, $A_{v}=-g_{m8}r_{o10}$ reveals the fundamental characteristic of the amplifier: $Q_{8}$ acts as the sole amplifying transistor, whereas $Q_{6}$ and $Q_{10}$ are primarily responsible for increasing impedance. The gain–bandwidth product (GBW) can be derived from $A_{v}$. The equivalent impedance at the output node is predominantly influenced by $r_{o10}$ and the compensation capacitor $c_{1}$, resulting in the dominant pole being defined as $w_{p}=1/r_{o10}c_{1}$.

For a single-pole amplifier, the unity-gain angular frequency satisfies $w_{t}=A_{v}w_{p}$. Substituting equations $A_{v}$ and $w_{p}=1/r_{o10}C_{1}$ yields

(11) $$w_{t}=\left(g_{m8}r_{o10}\right)\frac{1}{r_{o10}C_{1}}=\frac{g_{m8}}{C_{1}}$$

Therefore, the GBW is

(12) $$GBW=f_{t}=\frac{w_{t}}{2\pi }\;\cdot \;\frac{g_{m8}}{C_{1}}$$

This amplifier can be regarded as a single-pole amplifier, since a high impedance exists only at the output node (VO), forming the dominant pole with a relatively low pole frequency. The other nodes exhibit low impedance, leading to high-frequency poles whose effects can be neglected. The frequency response of this structure is clear, with a maximum phase shift of approximately 90°, resulting in a large phase margin that ensures inherently stable operation without oscillation. Moreover, the single-pole configuration greatly simplifies the frequency compensation design, making the gain–bandwidth product (GBW) easier to predict and control. Therefore, adopting a single-pole amplifier structure effectively enhances the stability and robustness of the BGR circuit under various load and supply conditions.

### 4.2. Driver Circuit Design

**Figure 6:**
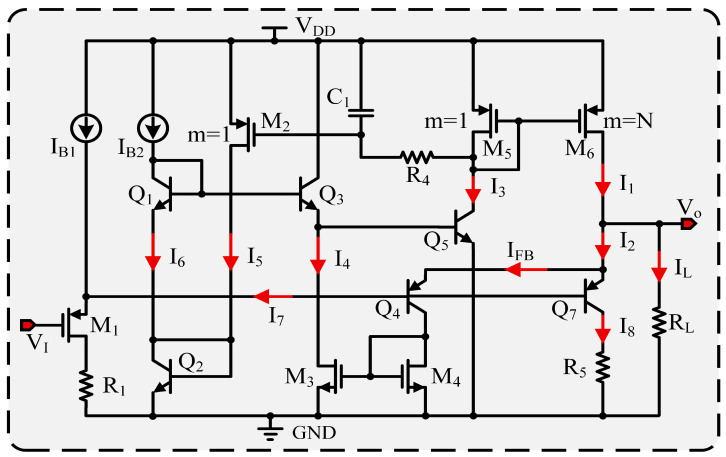
Driver circuit in the BGR.

The driver’s primary function in the design is to enhance its load-driving capacity while reducing its own current consumption. In the schematic, the current conversion section is substituted with a current source, and the essential circuitry of the driver is illustrated in Figure 6.

From Figure 6, the relationship between the output voltage and the input voltage of the driver can be expressed as

(13) $$V_{O}=V_{I}+V_{SG1}+V_{EB7}$$

The drain current of $M_{1}$ is set by $I_{B1}$ and $I_{7}$, where $I_{7}$ depends on the base currents of $Q_{4}$ and $Q_{7}$. Although $Q_{4}$ and $Q_{7}$ operate in the active region, their base currents change little; thus, $I_{1}$ is effectively constant and $V_{SG4}$ remains fixed. In Equation (13), the only varying term is $V_{EB7}$, which itself varies over a limited range. Consequently, the driver output $V_{O}$ does not provide voltage gain; it simply adds $V_{SG1}$ and $V_{EB7}$ to the input, raising it by about 1.2 V. In other words, the stage behaves as a voltage follower that automatically adjusts the output current of the power transistor $M_{6}$ to match the load.

In Figure 6, $M_{5}$ and $M_{6}$ create a current mirror configuration, where $M_{6}$’s multiplication factor is *N* times that of $M_{5}$, resulting in $I_{1}=NI_{3}$. The current $I_{3}$ is regulated by the base–emitter voltage of $Q_{5}$, which is interconnected with $Q_{1}$, $Q_{2}$ and $Q_{3}$. The relationship can be described as follows:

(14) $$V_{BE5}=V_{BE1}+V_{BE2}-V_{EB3}$$

Conversely, $V_{BE5}$ also serves as the drain voltage for the MOS transistor $M_{3}$, meaning $V_{BE5}=V_{DS3}$. Transistors $M_{3}$ and $M_{4}$ create a current mirror configuration, where the drain current of $M_{4}$ is regulated by the feedback current $I_{FB}$. This circuit features a dual feedback mechanism. To analyze the impact of current feedback, one must assume that both $V_{I}$ and $V_{O}$ are constant to establish a quiescent operating point. This point is defined when the load is disconnected ($R_{L}$ open), at which ($I_{2}=I_{1}$), as shown in Figure 6.

(15) $$I_{FB}=I_{2}-I_{8}=I_{1}-I_{8}$$

Under no-load conditions, the feedback current $I_{FB}$ drives the element labeled $I_{4}$ in the figure to its allowable maximum. At that point, $V_{BE5}$ sets the shunting of the current derived from $I_{1}$ through $Q_{7}$ so that it exactly equals $I_{FB}$, achieving balance. When a load $R_{L}$ is attached, a fraction of $I_{1}$ becomes $I_{L}$, which reduces $I_{2}$ and thus lowers $I_{FB}$. This increases $V_{DS3}$, which raises $I_{3}$ and, equivalently, lowers $V_{C5}$; the net effect is an increase in the $V_{SG}$ of $M_{6}$. Consequently, $I_{1}$ in Figure 6 increases, the output current grows, and the circuit attains a new equilibrium.

Intuitively, $M_{2}$ primarily controls the base–emitter voltage $V_{BE2}$ of $Q_{2}$ by adjusting the current $I_{5}$. When node $V_{C5}$ falls, the RC network formed by $R_{4}$ and $C_{1}$ produces a delay after which $M_{2}$’s gate–source voltage $V_{SG}$ increases. This increase raises $I_{5}$ and thus $V_{BE2}$. Via $Q_{1}$ and $Q_{3}$, the rise in $V_{BE2}$ drives $V_{BE5}$ upward, which in turn forces $V_{C5}$ to be lower, creating a positive feedback loop. The positive feedback can be interpreted another way: the $V_{BE5}$ given by Equation (14) acts as a limiting voltage that defines the allowable maximum, and $M_{2}$ automatically adjusts that limit. At a low-load current, $M_{2}$ keeps $V_{BE5}$ in a lower range to reduce static current; when load current increases, $M_{2}$ relaxes the limit so $V_{BE5}$ may rise to a higher value. To ensure that the adjustment of the limiting voltage lags behind changes in the load current $I_{L}$, a delay network comprising $R_{4}$ and $C_{1}$ is introduced. Although $M_{2}$ partially contributes to positive feedback, this effect is indirectly produced through changes in $V_{BE2}$, rendering it relatively weak and somewhat delayed compared to the feedback from $I_{FB}$. The base voltage of $Q_{5}$ is primarily controlled by the current amplifier formed by $I_{FB}$, $Q_{4}$, $M_{3}$ and $M_{4}$. Consequently, the system maintains an overall negative feedback characteristic.

Another component to highlight is the $Q_{7}$ branch shown in the figure, which regulates the range of the feedback current $I_{FB}$. Without $Q_{6}$, under no-load conditions, the output current $I_{1}$ of the power transistor $M_{6}$ would flow entirely into the feedback branch, preventing simultaneous stabilization of the operating points of $Q_{4}$ and $Q_{7}$. The $Q_{7}$ branch therefore both balances biasing across the transistor stages and gives the driver a modest current-sinking capability. For sinking currents in the 0–100 μA range, $Q_{5}$ and $Q_{4}$ remain in their active regions and thus satisfy the input–output voltage relationship specified by Equation (13).

**Figure 7:**
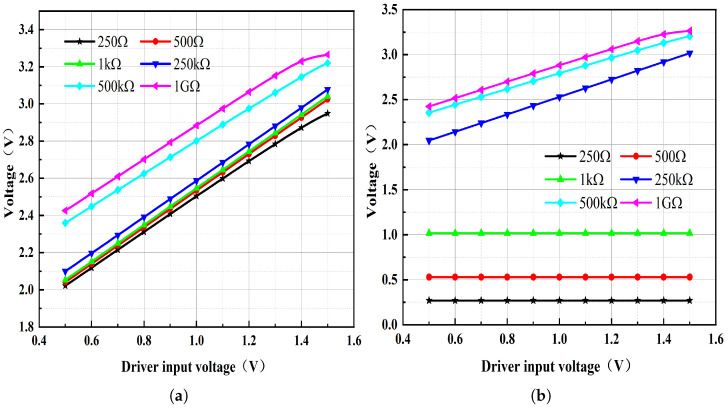
Driver output parameter sweep results as the driver input $V_{I}$ varies from 0.5 V to 1.5 V under different load resistances: (**a**) When $M_{2}$ is connected to $Q_{2}$. (**b**) When $M_{2}$ is disconnected from $Q_{2}$.

To further assess $M_{2}$’s effect on circuit performance, the driver input voltage ($V_{I}$) was swept from 0.5 to 1.5 V while the load resistor $R_{L}$ was treated as a variable in a DC sweep. Figure 6 plots the driver output voltage for $R_{L}$ values of 250 Ω, 500 Ω, 1 kΩ, 250 kΩ, 500 kΩ, and 1 GΩ. By disconnecting the link between $M_{2}$ and $Q_{2}$ and conducting the DC sweep analysis again, as illustrated in Figure 7, it becomes evident that after $M_{2}$ is disconnected, the driver’s output voltage drops significantly when $R_{L}$ is set to 250 Ω, 500 Ω and 1 kΩ. This observation suggests a substantial reduction in the circuit’s current-driving capability.

### 4.3. Frequency Compensation

Although the driver does not provide voltage gain, it still introduces an additional phase shift. Even though amplifier OP is a single-pole amplifier whose phase lag does not exceed 90° within its gain–bandwidth product, the overall phase shift of the system may exceed 180° when combined with the phase contribution of the driver, potentially leading to stability degradation. The phase shift introduced by the driver is strongly dependent on the load capacitance. Since the load capacitance varies over a wide range from 0.1 μF to 100 μF, the frequency response of the driver–load combination changes significantly, which increases the complexity of frequency compensation.

**Figure 8:**
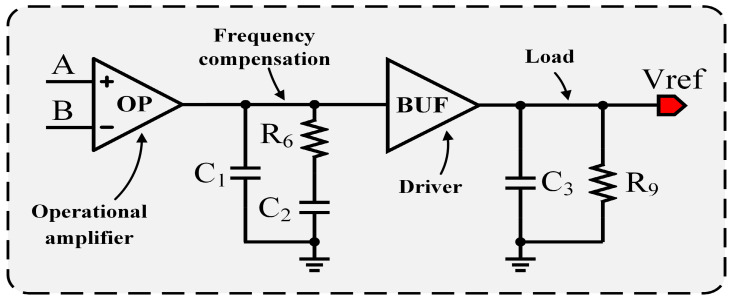
Schematic of the frequency compensation circuit.

After evaluating several compensation schemes, a basic zero-compensation approach was finally adopted. As shown in Figure 8, a compensation network is inserted between the amplifier and the driver. The fundamental idea of this network is to generate a zero using the $R_{6}$ and $C_{2}$ components, which is employed to counteract the pole introduced by the driver and the load capacitance. Meanwhile, capacitor $C_{1}$ is used to moderately reduce the bandwidth of amplifier OP, thereby improving the overall phase margin.

Because the load capacitance $C_{3}$ can be very large, the frequency of the second pole may be relatively low even though the output resistance of the driver is small. To effectively suppress the impact of this low-frequency pole, the zero generated by $R_{6}$ and $C_{2}$ must also be placed at a sufficiently low frequency. As a result, relatively large values of $R_{6}$ and $C_{2}$ are required.

### 4.4. Switched Resistor Array Design

**Figure 9:**
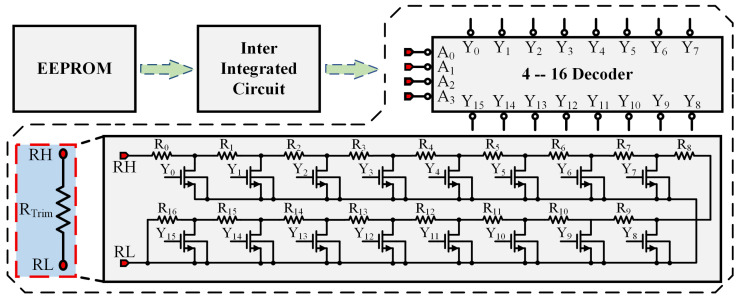
Switched Resistor Array.

Figure 9 shows the digital temperature compensation approach used in this design. Compensation is implemented by integrating a switched resistor array into the bandgap reference. The sensor’s measured temperature is used to correct the bandgap’s temperature-induced drift. Because the correction requires a large volume of data, storing it in fuses or on-chip OTP would occupy a substantial area. Consequently, this design stores the correction data in an off-chip EEPROM [29] accessed via the $I^{2}$C [30] interface shown in the figure.

To achieve a proper balance between system complexity and output accuracy, the output voltage of the BGR is trimmed at 10 °C intervals over the operating temperature range from −40 °C to 85 °C, resulting in a total of 14 trimming temperature points, with 85 °C treated as an additional independent trimming point. Prior to normal operation, post-layout simulations of the BGR output voltage are performed. At each trimming temperature point, the trimming codes A<3:0> and S<3:0> are adjusted to minimize the output voltage error, and the corresponding optimal codes are stored in separate memory locations of an off-chip EEPROM. It should be noted that identical values of A<3:0> and S<3:0> are assigned at the 20 °C and 30 °C trimming points to ensure a unique and well-defined trimming code combination at room temperature (27 °C). The trimming codes at the remaining temperature points are determined following the same consistency principle.

On chip power-up, the temperature sensor [31,32] produces control signals Ku<15:0> at various temperatures and forwards them to the control-logic module. When the control logic receives the Ku<15:0> pattern that matches a given tuning point, it engages the $I^{2}$C control module. That module reads the correction data A from the EEPROM into internal registers over $I^{2}$C. Finally, A<3:0> together with S<3:0> are applied to the switched resistor array to adjust the output voltage for the measured temperature [33]. The resistor trimming array consists of 17 series-connected resistor units, where the effective trimming resistance is adjusted by controlling the number of resistors connected in series. The 4-bit trimming codes, A<3:0> and S<3:0>, are fed into a 4-to-16 decoder, which generates the control signals for the switching MOS transistors. By selectively turning these switches on or off, different numbers of resistor units are connected in series, thereby precisely tuning the trimming resistance.

## 5. Simulation Results and Analysis

**Figure 10:**
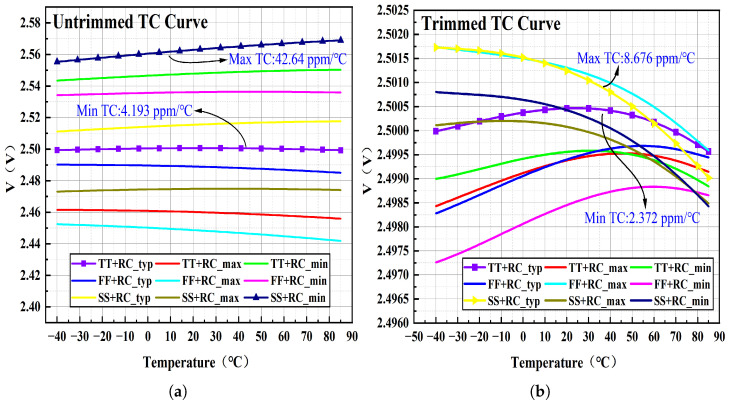
Temperature characteristics under nine process corners: (**a**) Temperature characteristics without trimming. (**b**) Temperature characteristics after trimming.

The BGR voltage source was implemented in a 0.18 μm 1P4M BCD process and simulated in Cadence Spectre. Figure 10 shows the temperature dependence of the bandgap output voltage ($V_{\mathrm{REF}}$) at a supply voltage of 3.3 V over a temperature range of −40 °C to 85 °C for nine process corners. Figure 10a reports the untrimmed temperature behavior, with a minimum TC of 4.193 ppm/°C and a maximum of 43.7 ppm/°C. Figure 10b reports the behavior after trimming, with a minimum TC of 2.372 ppm/°C and a maximum of 8.676 ppm/°C. These results indicate that resistor trimming substantially improves temperature stability, yielding excellent performance across all process corners.

In the circuit, noise on the power supply voltage is unavoidable. This noise can impact the output voltage of the reference source. The circuit’s ability to mitigate this effect is quantified by the PSRR, defined as follows:

(16) $$PSRR=20\log\frac{\Delta Vref}{\Delta V_{DD}}$$

**Figure 11:**
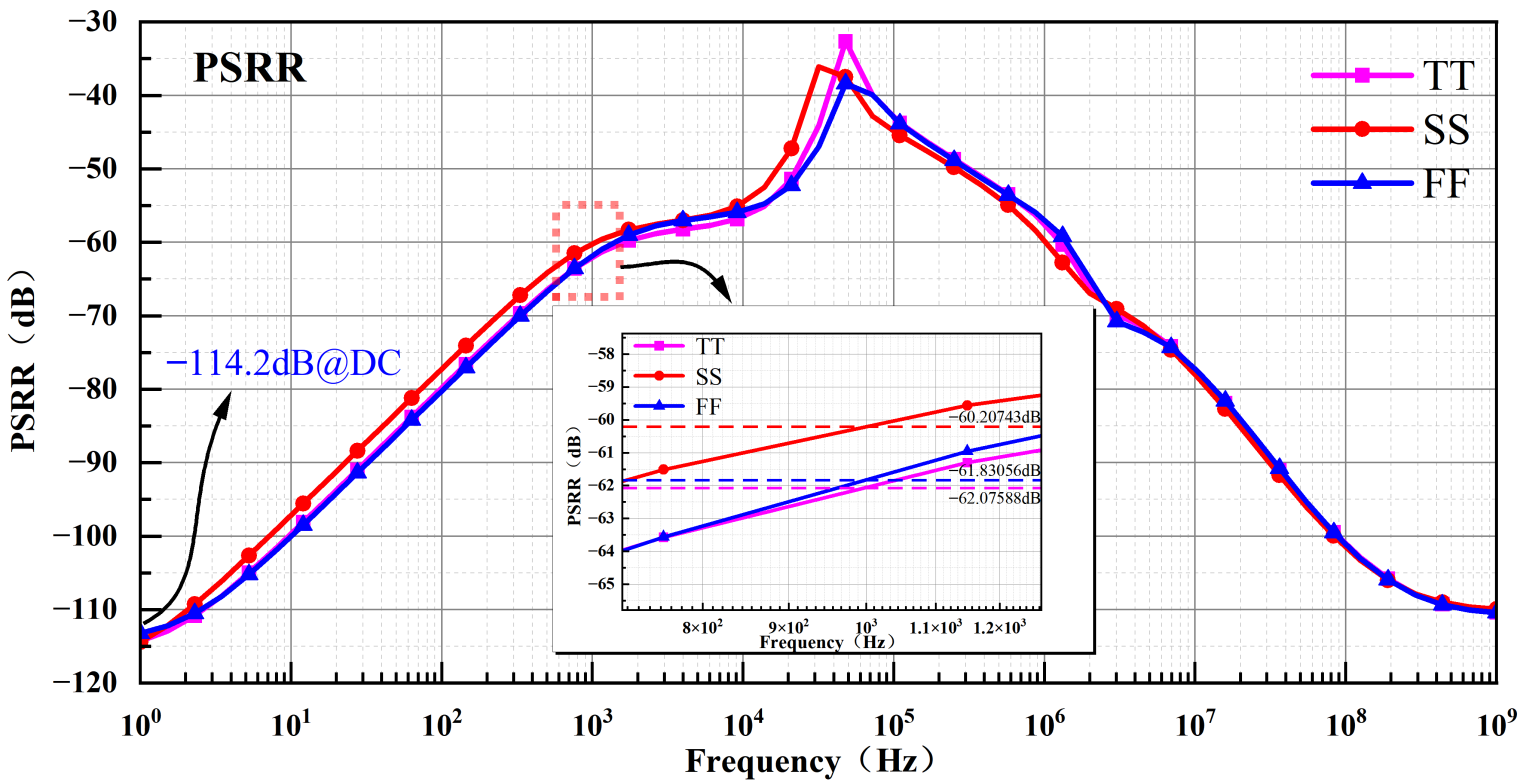
PSRR Simulation Results under TT, FF, and SS Process Corners.

In Equation (16), ΔVref denotes the change in reference output voltage caused by power-supply noise, and $\Delta V_{DD}$ denotes the magnitude of that noise (the ripple voltage). A larger PSRR therefore indicates that the circuit is less sensitive to supply fluctuations and better at suppressing power-supply disturbances. Figure 11 shows simulated PSRR curves for the BGR under the TT, SS, and FF process corners. At the TT corner, the PSRR is −113.97 dB at DC and −62.07588 dB at 1 kHz; at the SS corner, it is −114.55 dB at DC and −60.20743 dB at 1 kHz; and at the FF corner, it is −113.42 dB at DC and −61.83056 dB at 1 kHz. These results indicate that the proposed BGR provides excellent rejection of power-supply noise.

**Figure 12:**
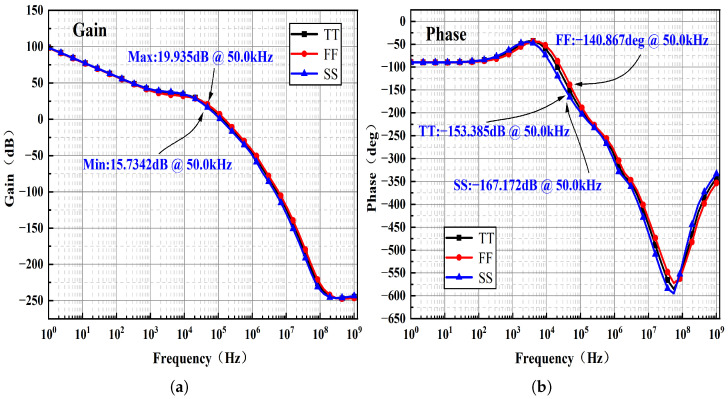
Combined frequency response of the amplifier, driver, and frequency compensation circuit: (**a**) Magnitude response. (**b**) Phase response.

Small-signal supply perturbations are coupled to the BGR output through multiple paths, including direct device parasitics and the biasing paths of the internal error amplifier. As illustrated in Figure 12, the combined frequency responses of the amplifier, driver, and frequency compensation circuit are simulated under TT, FF, and SS process corners. The results indicate that, at low frequencies, the high loop gain provided by the feedback loop effectively suppresses supply perturbations through negative feedback.

As the frequency increases, the dominant pole of the error amplifier causes a reduction in loop gain and introduces additional phase lag. When the accumulated phase shift approaches −180° and the loop gain decreases to approximately unity, the capability of the feedback loop to reject supply noise is significantly weakened, leading to a local peaking in the PSRR. Beyond this frequency range, the feedback loop becomes ineffective, and the PSRR is primarily governed by the intrinsic device-level rejection mechanisms.

The PSRR peak observed around 50 kHz coincides with the pronounced gain roll-off and the phase shift approaching −180° in the magnitude and phase response curves at this frequency. This behavior indicates insufficient loop gain and a markedly reduced ability to suppress supply perturbations.

Line regulation quantifies the sensitivity of the output reference voltage to changes in the supply voltage, indicating how supply voltage fluctuations affect the stability of the bandgap output. This measure is expressed in mV/V and can be represented by the following expression:

(17) $$LR=\frac{\Delta Vref}{\Delta V_{DD}}$$

**Figure 13:**
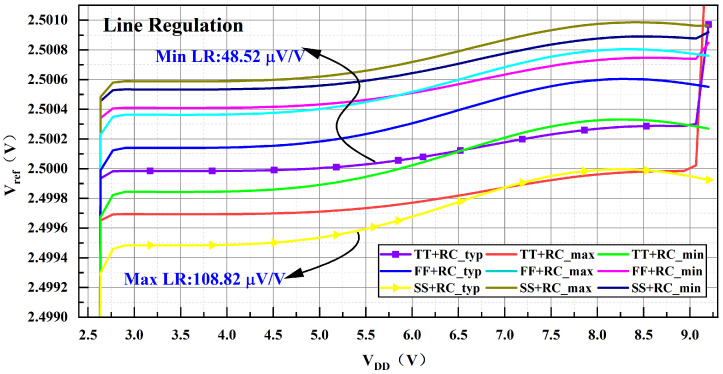
Line Regulation Simulation Results.

In Equation (17), $\Delta V_{DD}$ signifies the variation in supply voltage, while ΔVref indicates the change in reference voltage due to this variation. A smaller line regulation value suggests that the output reference voltage is less influenced by supply voltage fluctuations, allowing the reference to function stably across a broader supply voltage range. Figure 13 illustrates the bandgap output voltage variation concerning the supply voltage over a temperature span from −40 °C to 85 °C. The simulation results demonstrate that the circuit maintains linear regulation within the range of $2.7\;V\leq V_{DD}\leq 9.2\;V$. From the simulation results at different temperatures under the typical process corner, the minimum line regulation is 48.52 μV/V, while the maximum line regulation is 108.82 μV/V.

**Figure 14:**
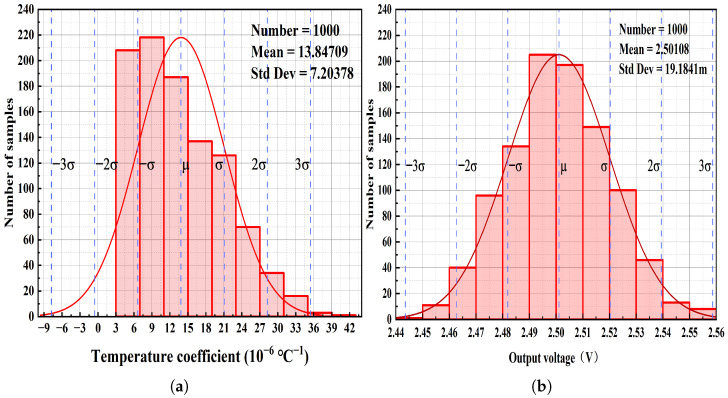
Monte carlo analysis: (**a**) Temperature coefficient of the BGR. (**b**) Output voltage of the BGR.

Under typical conditions, a Monte Carlo analysis using 1000 samples was conducted with the mismatch model for both the TC and the output voltage. The results are displayed in Figure 14a,b. Figure 14a shows that most temperature coefficients fall within the range of 3 $\times 10^{-6}$ to 15 $\times 10^{-6}$ °C^−1^, with an average of approximately 13.85 $\times 10^{-6}$ °C^−1^ and a standard deviation of about 7.2 $\times 10^{-6}$ °C^−1^. According to the Monte Carlo analysis results for the output voltage in Figure 14b, the output voltage is primarily distributed between 2.49 V and 2.51 V, with an average of 2.50108 V and a standard deviation of 19.1841 mV.

**Figure 15:**
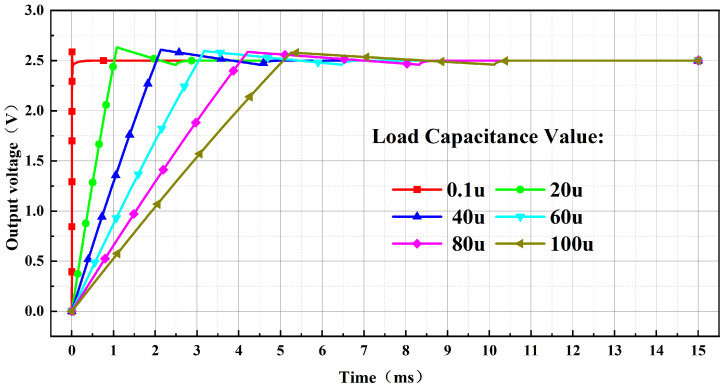
Simulation Results of Output Driving Capability with Load Capacitance.

Figure 15 presents the results of a load capacitance parameter sweep for the bandgap reference at a supply voltage of 3.3 V. The findings demonstrate that the output voltage of the BGR remains stable at 2.5 V across a load capacitance range of 0.1 μF to 100 μF.

**Figure 16:**
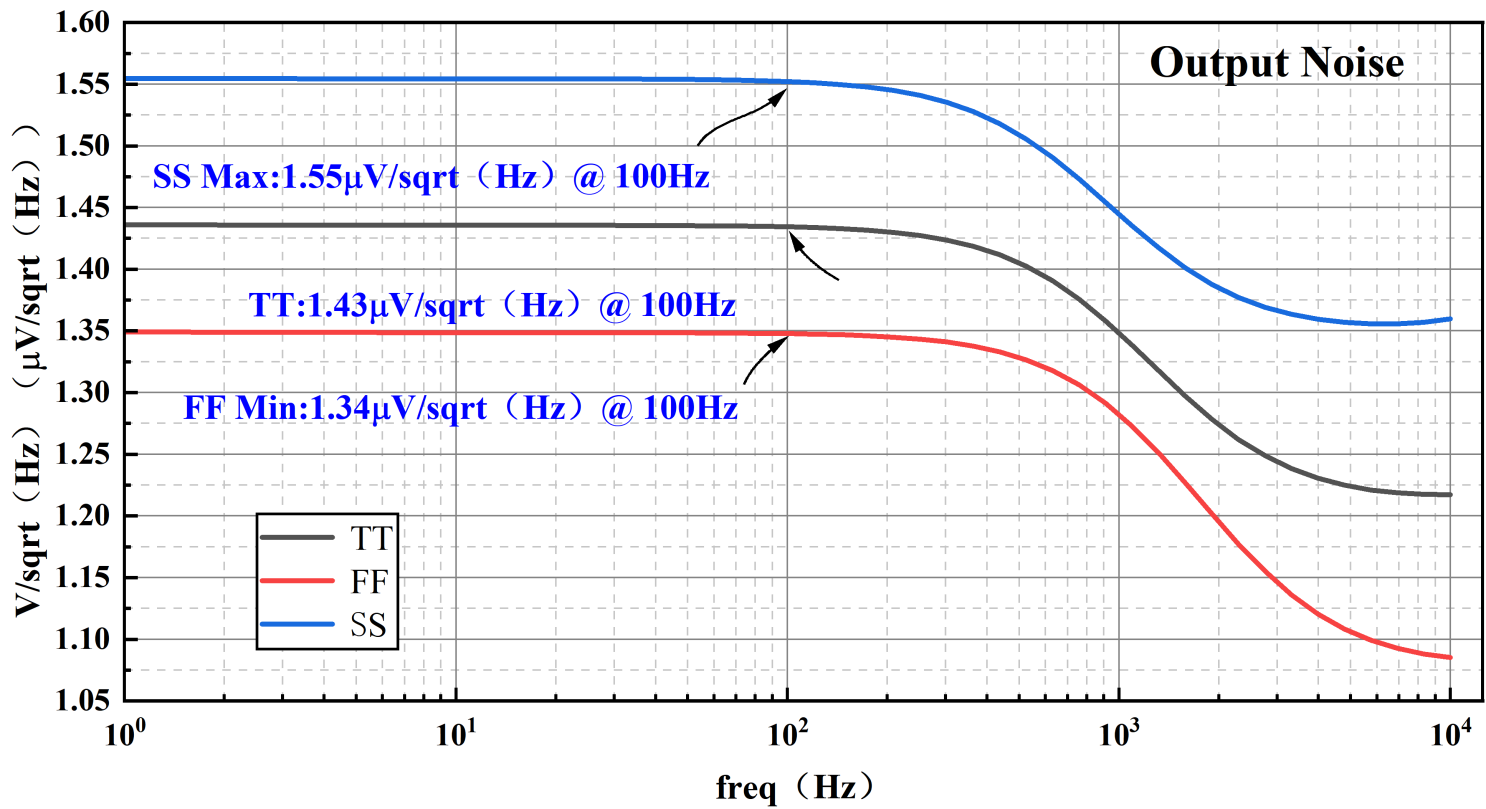
Equivalent output noise of the BGR.

Figure 16 illustrates the equivalent output noise of the proposed BGR under TT, FF, and SS process corners. At 100 Hz, the output reference noise is 1.34 μV/$\sqrt{\mathrm{Hz}}$ in the FF corner, 1.43 μV/$\sqrt{\mathrm{Hz}}$ in the TT corner, and 1.55 μV/$\sqrt{\mathrm{Hz}}$ in the SS corner.

**Figure 17:**
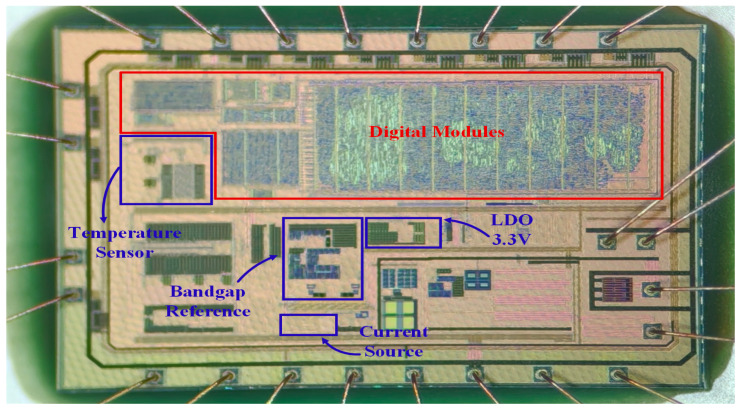
The 4–20 mA temperature transmitter chip including the BGR.

The complete 4–20 mA temperature transmitter chip, which integrates the BGR, temperature sensor, current source, and LDO circuits, is shown in Figure 17. The total chip area is 5.88 mm^2^, and the BGR occupies 0.12485 mm^2^. In Figure 17, the 3.3 V LDO supplies the voltage for the BGR, and the current source provides the reference current. The temperature sensor, in conjunction with the digital module, trims the BGR resistors.

Table 1 presents the performance of the proposed BGR alongside comparable designs and products. The proposed BGR offers high accuracy, strong PSRR, and robust load-driving capability, making it well suited for integration with high-performance ADCs, sensor interfaces, and power-management modules in industrial control loops, and for use as an LDO reference.

## 6. Conclusions

This design, implemented in a 180 nm BCD process, presents a high-precision BGR that combines a low TC, high PSRR, and strong load-driving capability. From an implementation perspective, the proposed BGR does not rely on any special devices or process options and is fully compatible with standard CMOS/BCD technologies. The use of a digitally assisted trimming scheme avoids complex analog calibration loops, which helps reduce test and trimming overhead. Moreover, the amplifier and driver circuits share a compact biasing structure, limiting additional device count. As a result, the proposed design achieves enhanced performance with moderate circuit complexity, making it cost-efficient for practical implementation. A low-offset amplifier structure is employed to improve PSRR effectively. A driver circuit is integrated into the core to stabilize the output under varying load conditions, thereby enhancing load-driving performance. A fine-tuning resistor array corrects deviations of the output voltage’s TC from the theoretical design value. Measurements show that the proposed BGR provides a stable 2.5 V output. After resistor trimming, the TC is reduced to 2.372 ppm/°C. The circuit also achieves excellent power-supply noise rejection, with a PSRR of −114 dB, and demonstrates superior load-driving performance.

## Figures and Tables

**Table 1:** Simulation performance of proposed BGR and comparison with other BGRs.

Parameter	This Work	[20]	[21]	[22]	[23]
Technology (nm)	180	180	180	180	180
Temperature Range (°C)	−40~85	−45~125	−45~125	−45~125	−40~150
Supply Voltage (V)	2.7~9.2	1.1~1.8	2.0~5.0	1.2	3.3
Current Consumption (μA)	69	60	–	400	120
Reference Voltage (V)	2.5	0.6	1.2	0.955	1.16
TC (ppm/°C)	2.372	2.4	32.7	23	5.78
PSRR (dB)	−114.2@DC	−76@DC	−85@100 Hz	−30@1 kHz	−82@10 kHz
Load Capacitance (μF)	0.1~100 μF	2.4 pF	–	–	–
Noise (μV/sqrt(Hz))	1.43 (1~100 Hz)	3.0 (0.1~10 Hz)	–	10@1 Hz	–
Line Regulation (μV/V)	48.52	30	580	500	300
Chip Area (mm^2^)	0.125	0.088	0.063	0.0121	0.08

## Data Availability

The data that support the findings of this study are available from the corresponding author upon reasonable request.

## References

[1] Azimi M., Habibi M., Crovetti P. (2024). A Two-Stage Sub-Threshold Voltage Reference Generator Using Body Bias Curvature Compensation for Improved Temperature Coefficient. Electronics.

[2] Gagliardi F., Bruschi P., Piotto M., Sakouhi S., Dei M. Single-Branch NMOS-Only Self-Cascoded Voltage Reference Operating Down to 0.28-V Supply. Proceedings of the 23rd IEEE Interregional NEWCAS Conference (NEWCAS).

[3] Xue W., Yu X., Zhang Y., Ming X., Fang J., Ren J. (2024). A 3.0-V 4.2-*μ*A 2.23-ppm/°C BGR with Cross-Connected NPNs and Base-Current Compensation. Microelectron. J..

[4] Park B., Ji Y., Sim J.Y. (2020). A 490-pW SAR Temperature Sensor with a Leakage-Based Bandgap-Vth Reference. IEEE Trans. Circuits Syst. II Express Briefs.

[5] Verma D., Shehzad K., Kim S.J., Pu Y.G., Yoo S.-S., Hwang K.C., Yang Y., Lee K.-Y. (2022). A Design of 10-Bit Asynchronous SAR ADC with an On-Chip Bandgap Reference Voltage Generator. Sensors.

[6] Tang Z., Liu Y., Chen P., Wang H., Yu X.P., Makinwa K.A., Tan N.N. (2024). A 14-b BW/Power Scalable Sensor Interface with a Dynamic Bandgap Reference. IEEE J. Solid-State Circuits.

[7] Zhu G., Yang Y., Zhang Q. (2019). A 4.6-ppm/°C High-Order Curvature Compensated Bandgap Reference for BMIC. IEEE Trans. Circuits Syst. II Express Briefs.

[8] Huang S., Li M., Li H., Yin P., Shu Z., Bermak A., Tang F. (2022). A Sub-1-ppm/°C Bandgap Voltage Reference with High-Order Temperature Compensation in 0.18-*μ*m CMOS Process. IEEE Trans. Circuits Syst. I Regul. Pap..

[9] Hunter B.L., Matthews J. (2017). A ±3 ppm/°C Single-Trim Switched Capacitor Bandgap Reference for Battery Monitoring Applications. IEEE Trans. Circuits Syst. I Regul. Pap..

[10] Jia S., Ye T., Xiao S. (2024). A 2.25 ppm/°C High-Order Temperature-Segmented Compensation Bandgap Reference. Electronics.

[11] Nageib E., Ibrahim S., Dessouky M. (2025). Resistor Variation Compensation for Enhanced Current Matching in Bandgap References. Electronics.

[12] Zhu X., Cui J., Li M., Zhang Y. (2023). A Piecewise Temperature-Curvature Compensation Bandgap with Internal Chopper-Stabilized Amplifiers. Microelectron. J..

[13] Zhuang H., Chen X., Zhang E., Li Q. (2025). High-Accuracy Bandgap Reference of <20 ppm/·C: A Review. Chips.

[14] Xu J., Wang Y., Wu M., Zhang R., Wei S., Zhang G., Yang C.F. (2019). A High-Accuracy Ultra-Low-Power Offset-Cancellation On–Off Bandgap Reference for Implantable Medical Electronics. Electronics.

[15] Ren J., Niu Y., Liu B., Li M., Bai Y., Chen Y. (2025). Design of an SAR-Assisted Offset-Calibrated Chopper CFIA for High-Precision 4–20 mA Transmitter Front Ends. Appl. Sci..

[16] Chen E., Wu T., Yu J., Yin L. (2023). A High-Precision Bandgap Reference with Chopper Stabilization and V-Curve Compensation Technique. Micromachines.

[17] Krolák D., Plojhar J., Horský P. (2020). An Automotive Low-Power EMC Robust Brokaw Bandgap Voltage Reference. IEEE Trans. Electromagn. Compat..

[18] Krolák D., Horský P. (2024). An EMI Susceptibility Improved, Wide Temperature Range Bandgap Voltage Reference. IEEE Trans. Electromagn. Compat..

[19] Yang F., Liu Y., Wang C., Zhao Y., Li Y. (2025). High Precision Radiation Resistant Bandgap Voltage Regulator for Aerospace Applications. IEEE Trans. Circuits Syst. I Regul. Pap..

[20] Liao X., Zhang Y., Zhang S., Liu L. (2024). A 3.0 *μ*V_rms_, 2.4 ppm/°C BGR with Feedback Coefficient Enhancement and Bowl-Shaped Curvature Compensation. IEEE Trans. Circuits Syst. I Regul. Pap..

[21] Huang W., Liu Y., Zhu Z. (2021). A Sub-200 nW All-in-One Bandgap Voltage and Current Reference Without Amplifiers. IEEE Trans. Circuits Syst. II Express Briefs.

[22] Khan A.A., Palani R. (2024). Analysis and Design of Low-Noise Voltage Regulator with Integrated Single BJT Bandgap Reference up to 10 mA Loads. IEEE Trans. Circuits Syst. II Express Briefs.

[23] Chen K., Petruzzi L., Hulfachor R., Onabajo M. (2021). A 1.16 V 5.8-to-13.5 ppm/°C Curvature-Compensated CMOS Bandgap Reference Circuit with a Shared Offset-Cancellation Method for Internal Amplifiers. IEEE J. Solid-State Circuits.

[24] Qi E., Fang C., Zhang Y., Cheng Y., Wang N. A wide input low quiescent current without operational amplifier bandgap reference circuit. Proceedings of the 2023 8th International Conference on Integrated Circuits and Microsystems (ICICM).

[25] Zhu G., Huang X., You Y., Li Y., Guo W., Zhu Z. (2025). Multi-Cell Battery Sensing and Protection IC with Integrated Low-Temperature-Drift Reference for Series Battery Pack Management. IEEE Trans. Circuits Syst. I Regul. Pap..

[26] Lee C.-F., U C.-W., Martins R.P., Lam C.S. (2024). 0.4-V Supply, 12-nW Reverse Bandgap Voltage Reference with Single BJT and Indirect Curvature Compensation. IEEE Trans. Circuits Syst. I Regul. Pap..

[27] Wang Z., Li Z., Xia X., Yu J., Zhou P., Chen J., Guo Y., Hong W. (2025). A 247–272-GHz SiGe Frequency Doubler with 8.2-dBm P_sat_ Enhanced by Optimized Fundamental Load Impedance and Hybrid-Mode Driver Amplifier. IEEE Trans. Microw. Theory Tech..

[28] Hou Y., Yu W., Yu Q., Wang B., Sun Y., Cheng W., Zhou M. (2021). A 56–161 GHz Common-Emitter Amplifier with 16.5 dB Gain Based on InP DHBT Process. Electronics.

[29] Kantor M., Molinazzi N., Shmilovich T., Krylov S. (2025). Low-Cost Polymeric Energy Harvester as Vibration Intensity Sensor. IEEE Sens. Lett..

[30] Albalooshi A., Jallad A.-H.M., Marpu P.R. (2023). Fault Analysis and Mitigation Techniques of the I^2^C Bus for Nanosatellite Missions. IEEE Access.

[31] Huang Z., Tang Z., Yu X.-P., Shi Z., Lin L., Tan N.N. (2021). A BJT-Based CMOS Temperature Sensor with Duty-Cycle-Modulated Output and ±0.5 °C (3*σ*) Inaccuracy from -40°C to 125 °C. IEEE Trans. Circuits Syst. II Express Briefs.

[32] Yousefzadeh B., Heidary Shalmany S., Makinwa K.A. (2017). A BJT-Based Temperature-to-Digital Converter with ±60 mK (3*σ*) Inaccuracy from -55°C to +125°C in 0.16-*μ*m CMOS. IEEE J. Solid-State Circuits.

[33] Ren J., Wang H., Li M., Liu B., Xiao J., Zhao W. (2025). A Wide-Input-Range LDO with High Output Accuracy Based on Digital Trimming Technique. Electronics.

